# The Recognition of Sweat Latent Fingerprints with Green-Emitting Carbon Dots

**DOI:** 10.3390/nano8080612

**Published:** 2018-08-12

**Authors:** Dan Zhao, Wenting Ma, Xincai Xiao

**Affiliations:** 1 School of Pharmaceutical Sciences, South-Central University for Nationalities, Wuhan 430074, China; wqzhdpai@163.com (D.Z.); tingwm1993@163.com (W.M.); 2 National Demonstration Center for Experimental Ethnopharmacology Education (South-Central University for Nationalities), Wuhan 430074, China

**Keywords:** green, carbon dots, sweat latent fingerprints, forensic science

## Abstract

The recognition of fingerprints has played an extremely important role in criminal investigations, due to its uniqueness. This paper reports on the recognition of sweat latent fingerprints using green-emitting, environment-friendly carbon dots prepared with DL-malic acid and ethylenediamine, and the exploration of impacting factors in the development process of fingerprints. The experiments showed that better fingerprint images could be obtained when the latent fingerprints are developed in green-emitting carbon dots with pH 9 for 30 min, at room temperature. The reported method was also effective for latent fingerprints on a variety of substrates, as well as for those water-immersed ones, where the developed fingerprint remained stable after long-term preservation. Furthermore, the fluorescent three-dimensional fingerprint image could provide direct and simple evidence on pressing habits. The objective of this paper was to present this method. The method may help to narrow the range of suspects during criminal investigations and in forensic science.

## 1. Introduction

The fingerprint of a human being, is influenced by heredity and environment, exhibits a property of uniqueness [1,2,3], and has already become important evidence in criminal investigations and convictions [2,4,5,6]. Generally, there are three common types of fingerprint evidence at crime scenes: Impression (or indented) fingerprints, visible (or patent) fingerprints, and latent fingerprints [6]. In most cases, the latent fingerprint is the most common, it is invisible to the naked eye [1,5,6], and can be further classified into bloody fingerprints and sweat fingerprints, according to the fingerprint residues. The bloody fingerprint, because of its easy and specific combination with chemical reagents on account of hemoglobin in the blood [7], is easily developed. The development of sweat fingerprints, however, still remains a problem. 

The fluorescence methods [8,9], are common methods for the development of fingerprints, including quantum dots (QDs) immersing and developing method [10], 1,8-diazafluoren-9-one treatment [6], etc. As a fluorescent nanomaterial, QDs have attracted widespread interest from researchers. Some teams have already applied oil-soluble CdS QDs and CdS/polyamidoamine, to the development of latent fingerprints on a variety of substrates based on the cyanoacrylate fuming method [11,12]. However, because of its complicated operation and the release of harmful cyanoacrylate ester during the process, this greatly limits its practical application. The development of fingerprints based on the direct immersion of the substrates into QDs developing solution, becomes an ideal substitute [10,13]. Researchers have already reported the acquisition of clear and detailed fingerprint images by immersing the substrates with latent fingerprints directly into water-soluble CdSe QDs [10] and CdTe QDs [14,15] solutions. Recently, Xu et al. [16] based on the N-L-Cys-capped Mn-doped ZnS QDs prepared by the modification of heavy metal-free Mn, realized the development of latent fingerprints on various objects. However, these methods still exhibit obvious shortcomings. Some of the developing solution contained Cd element, harmful to its users and the environment, and some required complicated preparation processes with strict synthesis environments and surface modifications. These problems greatly limit their applications in practical criminal investigations. 

The carbon dots (CDs), have the advantages of an easy preparation process and excellent biocompatibility [17,18,19], with potential applications in latent fingerprint development [20,21]. They have already exhibited incomparable merits in latent fingerprint development: (1) The sources of CDs are variable and environment friendly, making its preparation low cost; and (2) the CDs possess flexible plasticity in surface chemistry and biological properties. The required biometric features of fingerprints can be developed through a combination of the functional group on the surface of CDs and the fingerprint residues, because of the covalent coupling or electrostatic adsorption [22] through adjusting the environment of the solution. In 2018, Jiang et al. [20] reported the acquisition of white fluorescent fingerprint images by immersing the glass with the latent fingerprint into lipophilic white luminescent CDs which were dissolved in organic solvents. The researchers reported the development of a latent fingerprint via a spray method, using red luminescent CDs dissolved in 0.1 M hydrochloric acid [22]. Since solvents (organic solvents, 0.1 M pH 1 hydrochloric acid) are corrosive to most objects, such as plastics and leather, the method is harmful to the environment and its users. Furthermore, white fluorescent images exhibit low fluorescence intensity and vague outlines, greatly limiting its practical application. Wang et al. [23] synthesized excitation wavelength-dependent CDs, dominated by blue luminescent CDs using a spray and powder method to detect latent fingerprints on various objects, and obtained a blue fingerprint image. The fluorescence of CDs is mainly blue luminescence, which might be greatly limited by the excitation source specifically, the powder easily destroys the integrity of the fingerprint. It has strong background interference. Therefore, there is still a need to develop an environmentally friendly, low cost, and versatile fingerprint developing solution suitable for use at crime scenes, which ideally provides fingerprint images with a high definition and contrast. The green-emissioned CDs (G-CDs), due to its wide excitation spectrum, can be excited by selective wavelengths to distinguish the fluorescence of CDs from the background fluorescence, and thus enhance the contrast of the acquired images. G-CDs may have better results in fingerprint development. 

This paper reported on the development of latent sweat fingerprints with G-CDs as the developing solution, which is prepared using DL-malic acid and ethylenediamine. The impact of the pH values of G-CDs solution, as well as other experiment factors, including the substrates, the developing time, and the preserving time, have been systematically investigated. This method provides a simple and environment friendly way, for sweat fingerprint development, and has a promising outlook in criminal investigation and forensic science. 

## 2. Experimental Section

### 2.1. Chemicals and Apparatus

DL-malic acid (>99.0%) was obtained from Aladdin Chemistry Co., Ltd. (Shanghai, China); ethylenediamine (≥99.0%) was purchased from Sinopharm Chemical Reagent Co., Ltd. (Shanghai, China); tris-HCl buffer solutions with different pH values were prepared by dropwise addition into a concentrated hydrochloric acid solution (0.1 mol·L^−1^) or tris solution (0.05 mol·L^−1^), to required pH values. All chemicals used were of analytical grade or of the highest purity available. CDs with precursors citric acid as the carbon source and *N*-acetyl-l-cysteine as N and S dopant (B-CDs) had been synthesized [24]. Blue-emissioned CDs (P-B-CDs) had been synthesized from ethylene imine polymer and citric acid [25]. Yellow-emissioned CDs (Y-CDs) had been synthesized from o-phenylenediamine and *N,N*-dimethyl formamide [26]. Deionized water was prepared using a Milli-Q-RO4 water purification system (Millipore, Burlington, MA, USA).

UV-Vis absorption spectra were acquired with a Lambda-35 UV-Vis spectrophotometer, (PerkinElmer Company, Waltham, MA, USA) to determine the bandgap absorption of G-CDs. Fluorescence spectra were recorded on a LS55 spectrofluorometer (PerkinElmer Company, Waltham, MA, USA). Fourier transform infrared spectra were obtained on a Nicolet 6700 (FT-IR) spectrometer (Thermo Fisher Scientific, Waltham, MA, USA). The pH was monitored using a PHSJ-3F pH meter (Shanghai Precision Scientific Instrument Company, Shanghai, China). Images were obtained with a Gel Doc™ XR+ gel documentation system (Bio-Rad Laboratories, Inc., Hercules, CA, USA) or a Canon 5DII digital camera (Canon Inc., Tokyo, Japan). Images of latent fingerprints were lit on a ZF-1 three-use UV analyzer (Shanghai Jinpeng Analytical Instruments Co., Ltd., Shanghai, China). X-ray photoelectron spectroscopy (XPS) measurements were acquired with a VG Multilab 2000 X-ray photoelectron spectrometer (Thermo Electron Corporation, Waltham, MA, USA). A FELIX32 system (Photon Technology International, Birmingham, NJ, USA) was used to obtain the fluorescence intensity decay curves. All optical measurements were performed at room temperature. 

### 2.2. Synthesis of Green-Emissioned Carbon Dots (G-CDs)

According to Reference [27], G-CDs were synthesized, and the synthesis steps of G-CDs were improved. DL-malic acid (1.0 g) and ethylenediamine (0.4 mL) were added to deionized water (30 mL) to form a transparent solution. The solution was carried out under stirring for 10 min in N_2_ atmosphere. Subsequently, the mixture was heated in a teflon-equipped stainless-steel autoclave at 200 °C for 8 h. After naturally cooling to room temperature, the solution (*V*_G-CDs_:*V*_acetonitrile_ = 1:2) was centrifuged at 4000 rpm for 15 min, to remove the impurities. Finally, the purified G-CDs solution was diluted five times with deionized water, for further detection and use. 

### 2.3. Application in Latent Fingerprint Development

This paper selected fingerprints of two volunteers for fingerprint detection. Different fingers were cleaned with soap and water, and were dried naturally. The fingers were then gently rubbed across the forehead. Fingerprints were stamped with the appropriate strength. The latent fingermarks were deposited onto different objects, such as glass coverslips, sealed bags, transparent tapes, and tin foil papers. Subsequently, the latent fingermark objects were immersed in G-CDs solution, and then rinsed with deionized water and dried naturally. The fingerprint was placed in a darkroom, and excited with 365 nm UV light. The images of the fingerprint were taken using a Canon 5DII digital camera or gel imager. Fingerprint development can be found elsewhere in detail [10,13]. 

## 3. Results and Discussion

### 3.1. G-CDs Spectral Characterization and Application in Latent Fingerprint Development

#### 3.1.1. UV-Vis Spectrum and Fluorescence Spectrum

**Figure 1:**
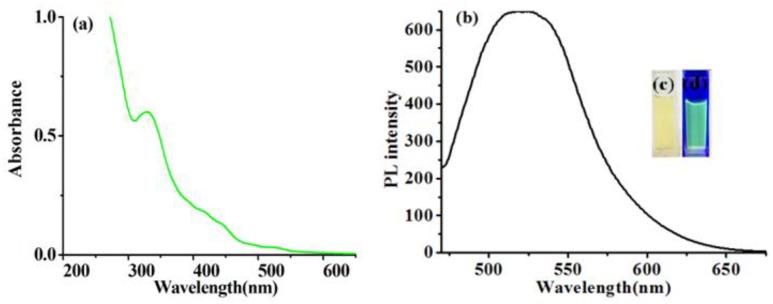
(**a**) UV-Vis spectrum of green-emissioned carbon dots (G-CDs) solution; (**b**) fluorescence spectrum of G-CDs solution; (**c**) daylight; (**d**) 440 nm blue light.

The G-CDs were synthesized via the hydrothermal method, from DL-malic acid with ethylenediamine. G-CDs exhibited one main UV-Vis absorption peak at 327 nm (Figure 1). The fluorescence spectrum of the G-CDs and images of different light sources, are shown in Figure 1. The excitation wavelength of prepared G-CDs was 460 nm, and the maximum emission wavelength was 520 nm. The fluorescence quantum yield was determined to be 19.6%, much higher than those reported in the literature [28]. The fluorescence decay curve for G-CDs is shown in Figure S1. The overall lifetime of G-CDs was 3.252 ns (λ_Ex_ = 481 nm, λ_Em_ = 542 nm).

When the excitation wavelength increased from 360 to 460 nm, the maximum emission peak shifted from 450 to 520 nm, together with the decrease of fluorescence intensity (Figure S2). The excitation wavelength dependent property, ensures a wide range of detection light (from UV to blue light) in practical applications when G-CDs are used as developing reagent in fingerprint development.

#### 3.1.2. Fingerprints Developed by Various QDs

Since the CDs exhibit obvious merits in their practical applications, such as simple preparation process, good biocompatibility and optical stability, low toxicity, etc., most reported CDs are blue-emitting. The fingerprint images developed by B-CDs, P-B-CDs, Y-CDs, and G-CDs (3.2 mg/mL) were compared.

**Figure 2:**
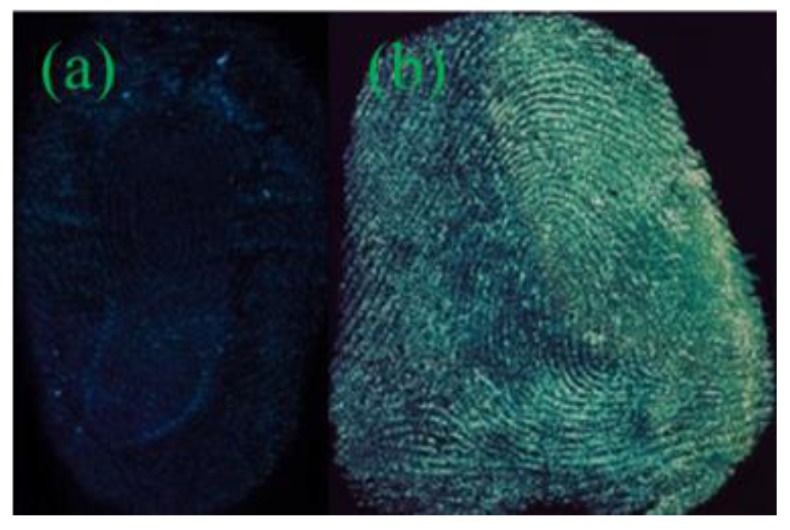
Diagrams of fingermarks on the tin foil paper after development using various quantum dots (QDs). (**a**) B-CDs; (**b**) G-CDs.

As shown in Figure S3, the image developed by Y-CDs shows no outline of the fingerprint. The fingerprint image developed by P-B-CDs can show a general outline, but the lines are vague. The image is low clarity with weak fluorescence intensity, almost without any practical value in fingerprint identification (Figure S3). As shown in Figure 2, the fingerprint developed by B-CDs possesses a clear outline, smooth lines with weak fluorescence intensity, and strong background interference. The maximum emission peak remained stable with sharp emission decrease when the excitation wavelength of B-CDs increased from 360 to 460 nm (Figure S2), showing that the change of excitation wavelength cannot alter the color of the fluorescence of B-CDs, and lead to weakened emission peak. The blue luminescence can be easily interfered by the background color of the substrates, and the acquired fingerprint image exhibits low resolution. The green luminescence of G-CDs, could provide fingerprint images with satisfactory contrast from the background colors, as well as clear and smooth fingerprint lines with sufficient details. The wide range of excitation wavelength, can ensure active avoidance from the background colors to reduce the background interference. The G-CDs were thus chosen as the developing reagent for further studies. 

### 3.2. The Optimization of Fingerprint Development in G-CDs Solution

#### 3.2.1. Fingerprints on Various Substrates

To assess the fingerprint acquisition ability of G-CDs on a variety of object surfaces, four substrates, being the sealed bags, glass coverslips, transparent tapes, and tin foil papers were selected in the fingerprint developing operations. 

**Figure 3:**
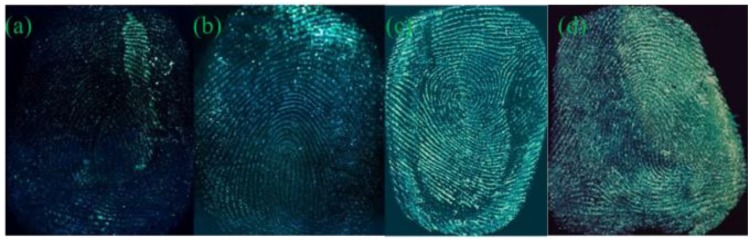
Fingermarks developed by G-CDs on various substrates. (**a**) sealed bag; (**b**) coverslip; (**c**) transparent tape; (**d**) tin foil paper.

Figure 3 presents the acquired images of latent fingerprints on these four object surfaces, developed by G-CDs solutions. The transparent tapes and tin foil papers presented excellent latent fingerprint images with obvious and coherent papillary ridges and depressed furrows lines, strong contrast against the background color distraction, and clear details. A wealth of authentication information for fingerprinting can be provided. The fingerprint on the surface of the coverslip showed better effects, but with relatively weak fluorescence intensity and fragility to external friction. The acquired latent fingerprint image on the sealed bag could be identified and had an obvious fingerprint profile, ridge lines were still readable, but with weaker fluorescence intensity. The smooth surfaces of the coverslip and sealed bag maintained fewer residues of sweat fingerprints, leading to weak fluorescence intensity on the images. Given the difference in the texture, color, and light reflection, G-CDs exhibited slight fluorescence color differences on these substrates. As the fingerprint on the tin foil paper was not easy to be glued and rubbed off, the tin foil was used as the subject for the following research. 

#### 3.2.2. Effect of pH

The fluorescence performance of G-CDs under different pH environments were examined before the assessments of pH to their developing effect. The fluorescence of G-CDs remained stable when the pH value ranges 2–12 and dropped dramatically when the pH surpassed 13, due to the instability of G-CDs in strong alkaline environments (Figure S4). Therefore, the assessment of developing performance of G-CDs to latent fingerprints, was set in moderate pH environments (pH values ranges from 2 to 12).

**Figure 4:**
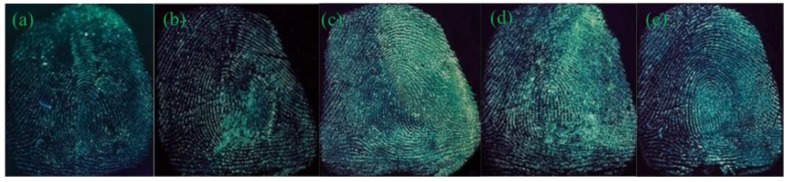
Fingermarks developed by G-CDs with different pH values. (**a**) pH = 6; (**b**) pH = 8; (**c**) pH = 9; (**d**) pH = 10; (**e**) pH = 12.

The latent fingerprints were developed according to the instructions stated in Section 2.3. The tin foil papers were immersed in G-CDs solutions with different pH values (pH = 6, 8, 9, 10, and 12), and the acquired fingerprint images are listed in Figure 4. The brightness of acquired images increased with the enhancement of pH value. Further increases of pH (>10) would lead to decreased florescence intensity, but the acquired images still had clear ridges and apparent details. That is the result of hydrolysis of fingerprint residue in acid or strong alkaline environments (pH = 6, 12) [10,13,29]. When pH reached 9–10, the acquired fingerprints exhibited strong fluorescence intensities, clear and coherent lines with uniform thickness, fine and smooth fingerprint profiles, and strong contrast from the background color distractions. The image reached their best when pH was set at 9. The carboxyl groups on the surface of G-CDs were negatively charged in alkaline solution [13], effectively preventing QDs from aggregation.

#### 3.2.3. The Impact of Developing Time

**Figure 5:**
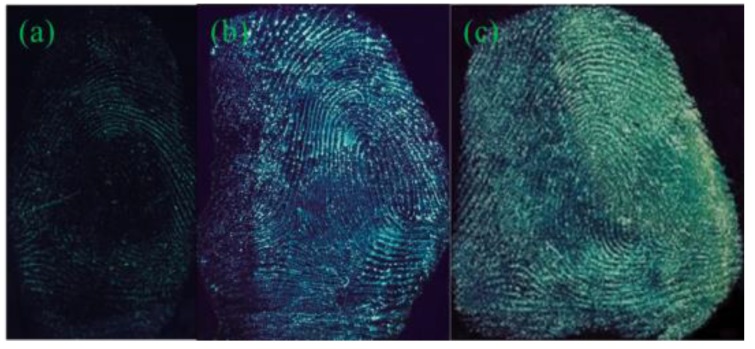
Fingermarks developed by G-CDs under various developing times. (**a**) 5 min; (**b**) 15 min; (**c**) 30 min.

The impact of developing time, that is, the time of substrates immersing in the G-CDs solution, was then investigated, with the latent fingerprints on the tin foil paper as the sample. Figure 5 presents the acquired fingerprint images, after the tin foil paper was immersed in the G-CDs solution for 5, 10, and 30 min under room temperature.

The fingerprint images acquired in our study presented clear and coherent ridges, detailed minutiae features, and strong contrasts after 15 min developing time. The prolonged developing time ensured better fingerprint images and strong fluorescence intensities. When the developing time reached 30 min, it achieved the best effect. Considering the timeliness of fingerprinting required by practical criminal investigation, 30 min is the optimal developing time. 

### 3.3. Application of Actual Samples

#### 3.3.1. Fingermarks Developed by G-CDs of Preserving Time

Forensic scientists always expect that the fingerprints developed by diverse techniques can maintain a good image after a long preservation time [29]. Therefore, the ability of evidence preservation is considered an important evaluating indicator to a fingerprint developing method. Transparent tape, as one of the most commonly used substrate for criminals, is chosen as the substrate for research.

**Figure 6:**
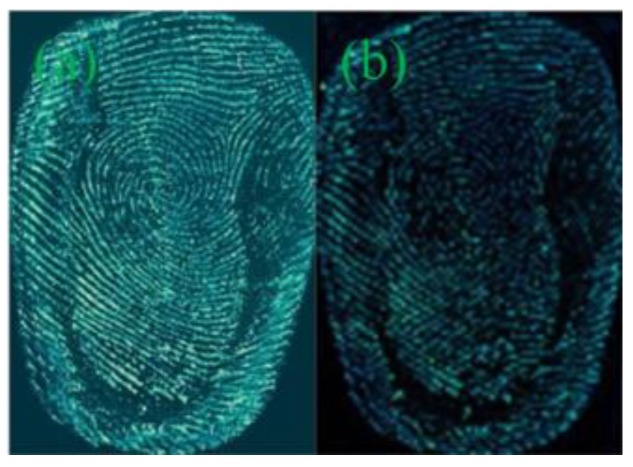
Fingermarks developed on the transparent tape by G-CDs of preserving time. (**a**) 1 day; (**b**) 60 days.

Figure 6 presents the fingerprint images on the transparent tape developed by G-CDs solution, after 60 days preservation. The fluorescence of the fingerprint is slightly weakened, but is still clear with detailed minutiae features and strong contrast against the background distractions, proving its effective ability in long-term preservation of evidence and practical meaning in their application in criminal investigations.

#### 3.3.2. The Development of Water-Soaked Latent Fingerprint 

Latent fingerprints may be found on items which have been accidentally or deliberately wetted on the crime scene [30]. This type of evidence is hardly visible and presents one of the difficulties in fingerprint developing technology. Therefore, the effective development of the water-soaked latent fingerprint is a hot spot in this field. Water-soaked objects are generally high-density ones, such as a knife and ax, so tin foil paper as an object was used for research. The latent fingerprint on the tin foil paper was soaked in water for four days, and then developed by G-CDs solution according to the standard operation.

**Figure 7:**
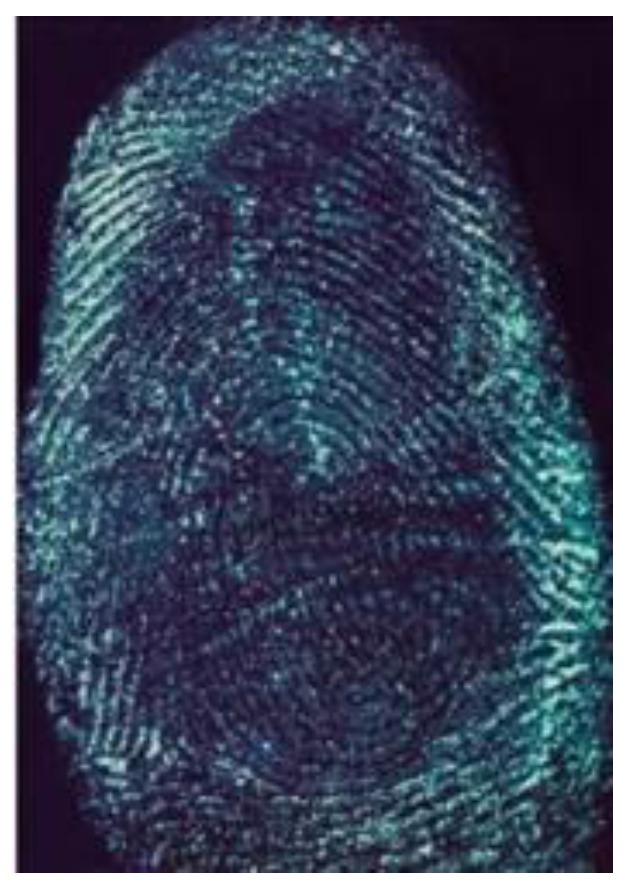
Fingermark developed by G-CDs on water-immersed substrate.

Figure 7 presents the acquired image of the water-soaked latent fingerprint. The fingerprint image was still clear in general profile with coherence in part of the papillary ridges, strong contrasts and enough minutiae features, but some of the lines were indistinct, as the result of the slow solution and diffusion of fingerprint materials on the papillary ridges of fingerprints. Some of the papillary ridges and depressed furrows exhibited adhesion but did not set critical impact on fingerprint identification.

#### 3.3.3. Three-Dimensional Image of Fingerprint developed in the G-CDs Solution

**Figure 8:**
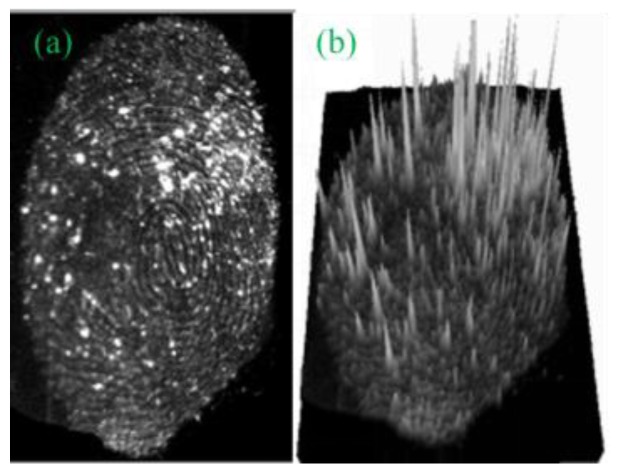
Fingermarks developed on transparent tape by G-CDs. (**a**) flat effect; (**b**) three-dimensional effect.

The possibility of a three-dimensional analysis of a latent fingerprint was then assessed. Figure 8 shows the developed latent fingerprint images on the transparent tape.

The experiments showed that only the appropriate press on the substrate, could produce the fingerprint with clear and coherent fingerprint details. Heavy press produced cohesion of lines and light press left limited fingerprint residue on the surface of the substrate, resulting in weak fluorescence intensity. Therefore, the fluorescence intensity of the acquired three-dimensional image, together with the visual minutiae features would be used to infer the customary press habit of the fingerprint owner, which is helpful for the narrowing down the range of suspects during criminal investigations.

### 3.4. Mechanism of Latent Fingerprints Development by G-CDs

**Figure 9:**
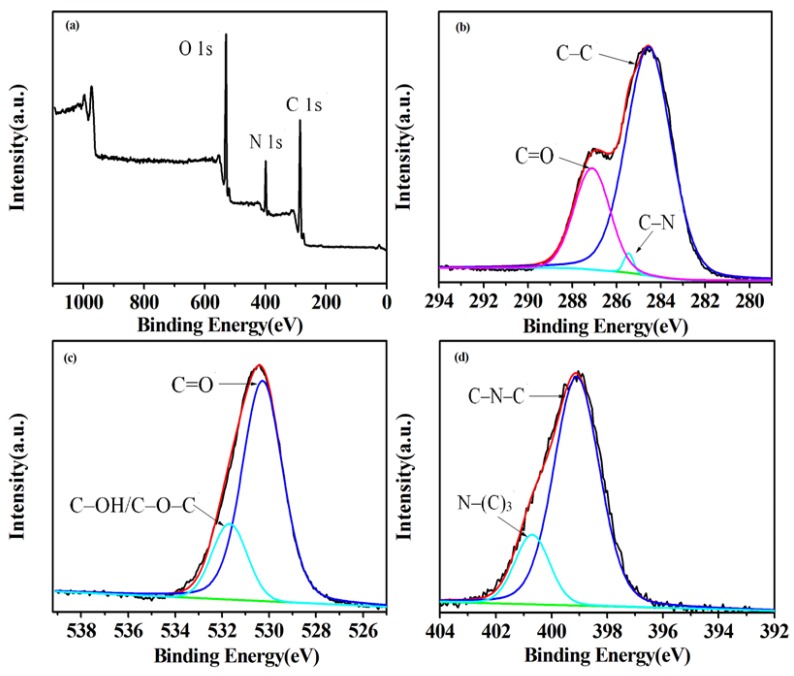
XPS spectra recorded from G-CDs. (**a**) Overall picture; (**b**) C 1s; (**c**) O 1s; (**d**) N 1s.

FTIR spectrum and XPS spectra were used to explore the mechanism of fingerprint development. As shown in Figure S5, the surface of G-CDs is rich in carbonyl, hydroxyl, and amino groups, according to the characteristic absorption peaks of some functional groups. The elemental analysis and surface composition for the resultant G-CDs were characterized by the XPS technique (Figure 9). Results showed that the as-prepared G-CDs contained C, N, and O three elements, at relative percentage composition 62.23%, 13% and 24.77%, respectively, indicating the presence of carbon as the major component along with other minor components like nitrogen and oxygen. The three peaks at about 284.6, 399.1, 530.4 eV shown in the XPS full scan spectrum (Figure 9a) could be attributed to C 1s, N 1s and O 1s, respectively. The C 1s spectrum (Figure 9b) could be deconvoluted into three peaks at 284.6, 285.4 and 287.1 eV, corresponding to C–C, C–N and C=O, respectively. The deconvoluted O 1s spectrum (Figure 9c) showed two speaks at about 530.3 and 531.7 eV, corresponding to C=O and C–OH/C–O–C bands, respectively. The deconvoluted N 1s spectrum (Figure 9d) exhibited a peak at 399.1 eV, corresponding to C–N–C and a peak at 400.7 eV, corresponding to N-(C)_3_ groups. All results from the FTIR spectrum and XPS studies confirm that the surface of the as-prepared G-CDs contained abundant carbonyl, hydroxyl, and amino groups. The fingerprint residue was made up of inorganic components (sodium chloride, water) and organic components (skin oil, amino acid, fatty acid, etc.). The carbonyl, hydroxyl, and amino groups on the surface of G-CDs would combine with the skin oil, amino acid, and fatty acid through electrostatic adhesion and chemical coupling effect; making G-CDs selectively deposit on the fingerprint lines and forming the fluorescence fingerprint images under ultraviolet light. 

## 4. Conclusions

This paper reported the realization of development of the latent fingerprint with green-emitting G-CDs as the developing reagent, which is one-step prepared using DL-malic acid and ethylenediamine. The experiments showed that the acquired fluorescence fingerprint images possessed clear and coherent ridges, enough minutiae features, and strong fluorescence intensity. The impact of a variety of experiment factors, including the pH values and the developing time, were systematically investigated. The results showed that fingerprints immersed in the G-CDs solution with pH 9 for 30 min, at room temperature obtained good effects. The reported latent fingerprint developing method can effectively develop the latent fingerprints on a variety of substrates, and also solve the problem of the development of water-immersed latent fingerprints. The developed fingerprint remained stable for long-term preservation. Furthermore, fluorescence three-dimensional fingerprint images could provide direct and simple evidence of the habit of pressing. This method helps to narrow the range of suspects during criminal investigations and forensic science. 

## Supplementary Materials

The following are available online at http://www.mdpi.com/2079-4991/8/8/612/s1, Figure S1: Fluorescence decay curve of green-emissioned carbon dots (G-CDs); Figure S2: Fluorescence spectra of G-CDs and B-CDs at different excitation wavelengths. (a) G-CDs; (b) B-CDs; Figure S3: Diagrams of fingermarks on the tin foil paper developed by various quantum dots (QDs). (a) Blue-emissioned carbon dots (P-B-CDs); (b) Yellow-emissioned carbon dots (Y-CDs); Figure S4: Fluorescence properties by G-CDs of various pH values; Figure S5: FTIR spectrum of the G-CDs.

## References

[1] Li F., Liu S.Q., Qi R.Y., Li H.R., Cui T.F. (2017). Effective visualization of latent fingerprints with red fluorescent La_2_ (MoO_4_)_3_:Eu^3+^ microcrystals. J. Alloys Compd..

[2] Ambadiyil S., Prakash D., Sheeja M.K., Mahadevan Pillai V.P. (2017). Secure storage and analysis of fingerprints for criminal investigation using holographic techniques. Mater. Today Proc..

[3] Li Y.Q., Xu C.Y., Shu C., Hou X.D., Wu P. (2017). Simultaneous extraction of level 2 and level 3 characteristics from latent fingerprints imaged with quantum dots for improved fingerprint analysis. Chin. Chem. Lett..

[4] Milasinovic N. (2016). Polymers in criminalistics: Latent fingerprint detection and enhancement: From idea to practical application. Nauka Bezb. Polic..

[5] Bailey M.J., Ismail M., Bleay S., Bright N., Levin Elad M., Cohen Y., Geller B., Everson D., Costa C., Webb R.P. (2013). Enhanced imaging of developed fingerprints using mass spectrometry imaging. Analyst.

[6] Wang M., Li M., Yu A., Zhu Y., Yang M.Y., Mao C.B. (2017). Fluorescent nanomaterials for the development of latent fingerprints in forensic sciences. Adv. Funct. Mater..

[7] Petretei D., Angyal M. (2015). Recovering bloody fingerprints from skin. J. Forensic Identif..

[8] Song Z.P., Li Z.H., Lin L.H., Zhang Y.F., Lin T.R., Chen L., Cai Z., Lin S., Guo L.Q., Fu F.F. (2017). Phenyl-doped graphitic carbon nitride: Photoluminescence mechanism and latent fingerprint imaging. Nanoscale.

[9] Li F., Li H.R., Cui T.F. (2017). One-step synthesis of solid state luminescent carbon-based silica nanohybrids for imaging of latent fingerprints. Optmat.

[10] Wang Y.F., Yang R.Q., Shi Z.X., Liu J.J., Zhao K., Wang Y.J. (2014). The effectiveness of CdSe nanoparticle suspension for developing latent fingermarks. J. Saudi Chem. Soc..

[11] Menzel E.R., Savoy S.M., Ulvick S.J., Cheng K.H., Murdock R.H., Sudduth M.R. (2000). Photoluminescent semiconductor nanocrystals for fingerprint detection. J. Forensic Sci..

[12] Jin Y.J., Luo Y.J., Li G.P., Li J., Wang Y.F., Yang R.Q., Lu W.T. (2008). Application of photoluminescent CdS/PAMAM nanocomposites in fingerprint detection. Forensic Sci. Int..

[13] Wang Y.F., Yang R.Q., Wang Y.J., Shi Z.X., Liu J.J. (2009). Application of CdSe nanoparticle suspension for developing latent fingermarks on the sticky side of adhesives. Forensic Sci. Int..

[14] Liu J.J., Shi Z.X., Yu Y.C., Yang R.Q., Zuo S.L. (2010). Water-soluble multicolored fluorescent CdTe quantum dots: Synthesis and application for fingerprint developing. J. Colloid Interface Sci..

[15] Gao F., Han J.X., Zhang J., Li Q., Sun X.F., Zheng J.C., Bao L.R., Li X., Liu Z.L. (2011). The synthesis of newly modified CdTe quantum dots and their application for improvement of latent fingerprint detection. Nanotechnology.

[16] Xu C.Y., Zhou R.H., He W.W., Wu L., Wu P., Hou X.D. (2014). Fast imaging of eccrine latent fingerprints with nontoxic Mn-doped ZnS QDs. Anal. Chem..

[17] Shi Y.L., Wei S., Gao Z.Q. (2015). Carbon quantum dots and their applications. Chem. Soc. Rev..

[18] Ding H., Yu S.B., Wei J.S., Xiong H.M. (2015). Full-color light-emitting carbon dots with a surface-state-controlled luminescence mechanism. ACS Nano.

[19] Zhao S.J., Lan M.H., Zhu X.Y., Xue H.T., Ng T.W., Meng X.M., Lee C.S., Wang P.F., Zhang W.J. (2015). Green synthesis of bifunctional fluorescent carbon dots from garlic for cellular imaging and free radical scavenging. ACS Appl. Mater. Interfaces.

[20] Jiang B.P., Yu Y.X., Guo X.L., Ding Z.Y., Zhou B., Liang H., Shen X.C. (2018). White-emitting carbon dots with long alkyl-chain structure: Effective inhibition of aggregation caused quenching effect for label-free imaging of latent fingerprint. Carbon.

[21] Khan W.U., Wang D.Y., Zhang W., Tang Z.B., Ma X.L., Ding X., Du S.S., Wang Y.H. (2017). High quantum yield green-emitting carbon dots for Fe (ІІІ) detection, biocompatible fluorescent ink and cellular imaging. Sci. Rep..

[22] Chen J., Wei J.S., Zhang P., Niu X.Q., Zhao W., Zhu Z.Y., Ding H., Xiong H.M. (2017). Red-emissive carbon dots for fingerprints detection by spray method: Coffee ring effect and unquenched fluorescence in drying process. ACS Appl. Mater. Interfaces.

[23] Wang C., Zhou J.D., Lu L.L., Song Q.J. (2018). Rapid visualization of latent fingerprints with color-tunable solid fluorescent carbon dots. Part. Part. Syst. Charact..

[24] Gao F., Ma S.Y., Li J., Dai K., Xiao X.C., Zhao D., Gong W.F. (2017). Rational design of high quality citric acid-derived carbon dots by selecting efficient chemical structure motifs. Carbon.

[25] Li J.Y., Liu Y., Shu Q.W., Liang J.M., Zhang F., Chen X.P., Deng X.Y., Swihart M.T., Tan K.J. (2017). One-pot hydrothermal synthesis of carbon dots with efficient up-and down-converted photoluminescence for the sensitive detection of morin in a dual-readout assay. Langmuir.

[26] Lin S., Cheng Y.Z., Lin C., Fang J.L., Xiang W.D., Liang X.J. (2018). Carbon nanodots with intense emission from green to red and their multifunctional applications. J. Alloys Compd..

[27] Pang S.B., Zhang Y., Wu C.K., Feng S.L. (2016). Fluorescent carbon dots sensor for highly sensitive detection of guanine. Sens. Actuators B Chem..

[28] Xiao D.L., Yuan D.H., He H., Gao M.M. (2013). Microwave assisted one-step green synthesis of fluorescent carbon nanoparticles from ionic liquids and their application as novel fluorescence probe for quercetin determination. J. Lumin..

[29] Cai K.Y., Yang R.Q., Wang Y.J., Yu X.J., Liu J.J. (2013). Super fast detection of latent fingerprints with water soluble CdTe quantum dots. Forensic Sci. Int..

[30] Rohatgi R., Sodhi G.S., Kapoor A.K. (2015). Small particle reagent based on crystal violet dye for developing latent fingerprints on non-porous wet surfaces. Egypt. J. Forensic Sci..

