# Supplementary material for: The Recognition of Sweat Latent Fingerprints with Green-Emitting Carbon Dots
Source: Nanomaterials (Basel). 2018 Aug 12;8(8):612. doi: 10.3390/nano8080612 (PMC6116216; doi:10.3390/nano8080612)
Supplement: Supplementary file 1 [file nanomaterials-08-00612-s001.pdf]

Supplementary Material

The Recognition of Sweat Latent Fingerprints with Green-emitting Carbon Dots

Dan Zhao[1,2,†], Wenting Ma[1,2,†], Xincai Xiao[1,2,*]

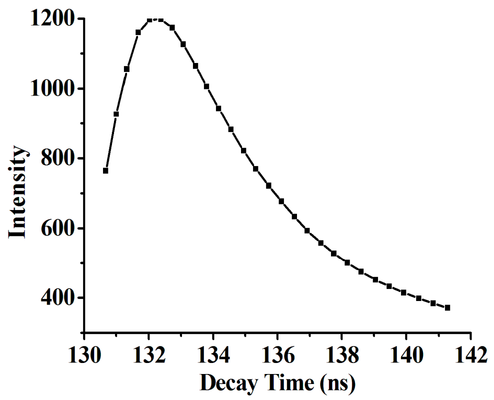

Figure S1. Fluorescence decay curve of green-emissioned carbon dots (G-CDs).

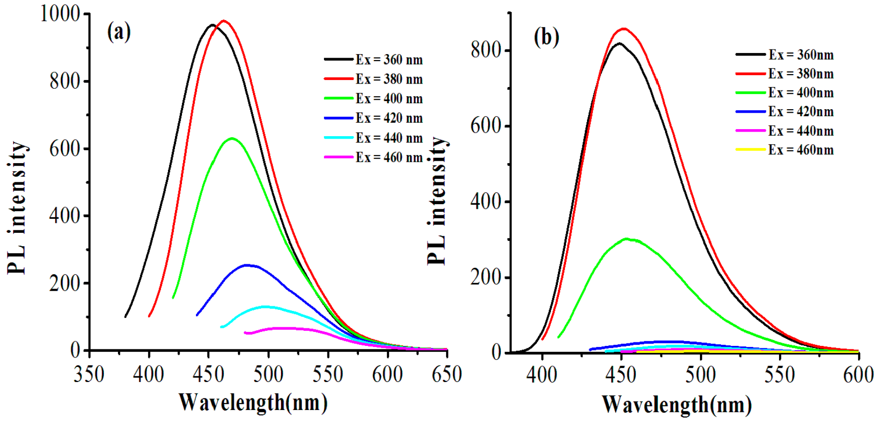

Figure S2. Fluorescence spectra of G-CDs and B-CDs at different excitation wavelengths. (a) G-CDs; (b) B-CDs.

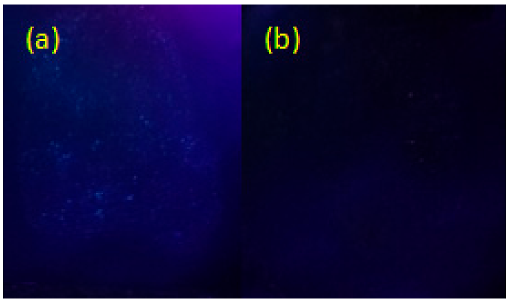

Figure S3. Diagrams of fingermarks on the tin foil paper developed by various quantum dots (QDs). (**a**) Blue-emissioned carbon dots (P-B-CDs); (**b**) Yellow-emissioned carbon dots (Y-CDs).

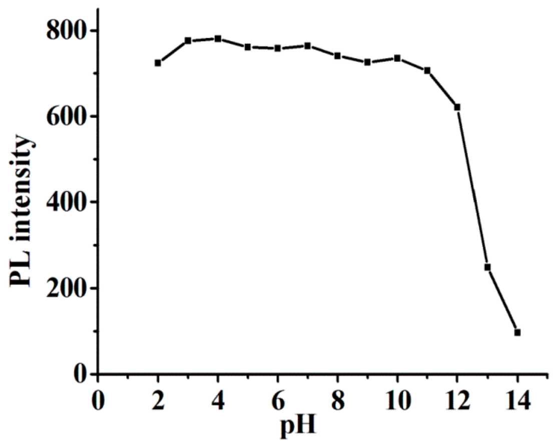

Figure S4. Fluorescence properties by G-CDs of various pH values.

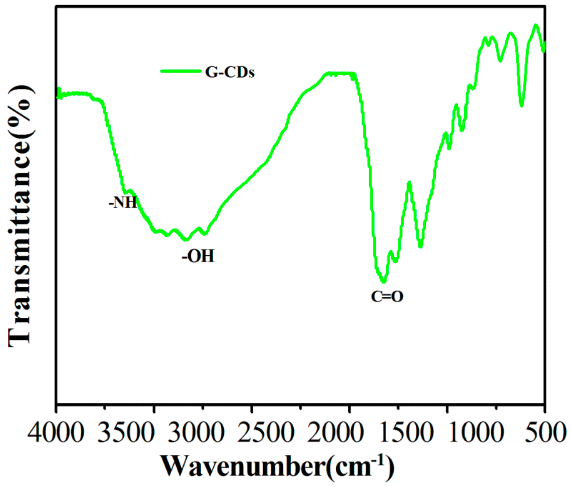

Figure S5. FTIR spectrum of the G-CDs.
